# Updates on Therapy Options in Fit and Unfit Patients with Newly Diagnosed AML

**DOI:** 10.1007/s11864-025-01351-3

**Published:** 2025-08-23

**Authors:** Gray H. Magee, Michael R. Grunwald

**Affiliations:** 1 Atrium Health Levine Cancer Institute, 1021 Morehead Medical Drive, LCI Building 2, Charlotte, NC 28204 USA; 2 Division of Hematology, Atrium Health Levine Cancer Institute and Wake Forest University School of Medicine, 1021 Morehead Medical Drive, LCI Building 2, Suite 60100, Charlotte, NC 28204 USA

**Keywords:** Acute myeloid leukemia, Frontline therapy, Targeted therapy, KMT2A rearrangement, FLT3 mutation, IDH mutation

## Abstract

The integration of next-generation sequencing (NGS) and advanced cytogenetic diagnostics into routine clinical practice is reshaping frontline treatment of acute myeloid leukemia (AML) in both fit and unfit patients. Molecular profiling now enables personalized treatment strategies, particularly for patients harboring mutations in *FLT3*, *IDH1*, *IDH2*, *KMT2A*, and *NPM1*. Small molecule inhibitors, first reserved for relapsed/refractory disease, are increasingly used in the upfront setting. However, universal NGS testing at diagnosis is critical to identify eligible patients for these targeted therapies. In patients lacking actionable mutations, treatment can still be refined using karyotypic abnormalities or high-risk features suggestive of antecedent MDS. In our practice, we continue to use 7 + 3 induction for fit patients, adding midostaurin or quizartinib for *FLT3*-mutated AML, or gemtuzumab ozogamicin for core binding factor (CBF) AML expressing CD33. For patients with therapy-related AML or AML with myelodysplasia-related changes, CPX-351 is our standard induction approach. For unfit patients, we generally offer hypomethylating agents with venetoclax. In the presence of *IDH1* mutations, we consider azacitidine combined with ivosidenib. If venetoclax is contraindicated or not tolerated, targeted therapies such as gilteritinib, ivosidenib, or enasidenib may be appropriate based on mutation profile. However, we try to identify clinical trials for all our patients at diagnosis. One of the more exciting recent developments is the emergence of menin inhibitors for patients with *KMT2A* rearrangements or *NPM1* mutations. While several agents have received FDA approval or breakthrough status in the relapsed/refractory setting, they are now being actively studied as frontline options with promising results. When feasible, clinical trial enrollment should be considered for newly diagnosed patients with these alterations. As the therapeutic landscape for AML continues to evolve, timely molecular characterization is more essential than ever to optimize outcomes and select the most appropriate frontline strategy.

## Introduction

Acute Myeloid Leukemia (AML) is a clinically heterogenous disease characterized by uncontrolled proliferation of clonal myeloid progenitor cells in the bone marrow with associated block in maturation and differentiation in the myeloid lineage, often resulting in bone marrow failure. AML accounts for approximately 20–25% of new leukemia cases in the United States with a projected 22,010 new cases in the United States in 2025 [1]. Prognosis for a patient newly diagnosed with AML largely depends on the cytogenetic aberrations that led to neoplasia as well as the patient’s ability to tolerate intensive chemotherapy due to age and comorbid conditions [2–4].

For newly diagnosed patients, intensive induction with continuous cytarabine infusion for seven days with concurrent anthracycline chemotherapy with either 3 consecutive days of daunorubicin or idarubicin (“7 + 3”) has been the backbone of upfront treatment [5, 6]. Patients who achieve remission can then receive consolidation treatment with either sequential cycles of high dose cytarabine (HiDAC) or allogeneic stem cell transplant if an appropriate bone marrow donor is found [7–11].

However, “unfit” patients who are not candidates to receive 7 + 3 and/or allogeneic stem cell transplant have historically had limited options for therapy and previously relied on low dose chemotherapy with suboptimal efficacy or best supportive care with intermittent blood product transfusions and infectious prophylaxis if indicated. This landscape has changed in the 21st century with the advent of hypomethylating agents (HMAs) as well as the oral BCL2 inhibitor venetoclax, as these agents maintain much of the efficacy of traditional cytotoxic chemotherapy with a better side effect profile that is more tolerable in older patients and patients with significant comorbidities [12–14]. HMAs are relatively newer therapies initially developed for treatment of myelodysplastic syndrome (MDS). Azacitidine and decitabine are pyrimidine nucleoside analogues that primarily act by inhibiting DNA methyltransferase, thereby altering gene transcription and expression of oncogenes via hypomethylation [15]. However, there is also direct cytotoxicity via incorporation into DNA and RNA similar to other nucleoside analogues [15].

Additionally, the introduction of next-generation sequencing (NGS) testing into the clinical space has led to developments in prognostication of newly diagnosed AML as well as developments in targeted therapies, leveraging specific genetic mutations known to be associated with AML such as *FLT3*, *IDH1*, *IDH2*, and recently *KMT2A* [16]. These advances have led to new options for upfront treatment of AML, both for fit patients who are candidates for intensive chemotherapy, and those who are unfit.

In this review, we discuss new and emerging therapies for newly diagnosed patients with AML. This summary includes both mutation-agnostic treatments as well as targeted therapies.

## Options for Intensive Treatment-Eligible Patients

### AML without Targetable Genetic Aberration

Since the initial study using the regimen in 1973, 7 + 3 has remained the induction backbone for fit patients with newly diagnosed AML [5]. Recent studies have demonstrated an overall response rate (ORR) of approximately 70–75%, a 3 year event-free survival (EFS) of 35–50%, and a 3 year overall survival (OS) of 50–60% [17, 18]. Therefore, there has been a desire to layer additional treatments onto the 7 + 3 backbone to improve response rates and survival.

### Gemtuzumab Ozogamicin

Gemtuzumab ozogamicin (GO) is an intravenous antibody–drug conjugate with a monoclonal antibody directed at CD33, which is commonly expressed on leukemic myeloblasts and not on pluripotent stem cells or mature myeloid cells, combined with a calicheamicin derivative as the payload [19]. GO was first approved by the Federal Drug Administration (FDA) in 2000 for use in the relapse setting in patients with CD33 + expressing AML who are 60 years of age or older on a dosing schedule of 9mg/m2 on day 1 and day 15 [20]. However, the agent was voluntarily withdrawn from the market in 2010 after a cooperative group trial studying the role of GO in combination with 7 + 3 demonstrated that GO added to standard induction therapy did not provide a survival benefit and resulted in increased early mortality (5% vs 1%) as well as a significantly increased risk of veno-occlusive disease (VOD) compared to placebo, which seemed to be magnified in patients later receiving allogeneic transplant [21].

GO was later studied again in the induction setting combined with 7 + 3 at an alternative dosing schedule (3mg/m2 on D1, D4, D7), which showed an initial significant difference in 2 year EFS (40.8% vs 17.1%). However, the study failed to demonstrate a significant difference in OS (53.2% vs 41.9%). Despite no statistically significant differences in early death, there was a significant difference in rates of VOD and thrombocytopenia in patients receiving GO, similarly to the cooperative group trial that led to voluntary withdrawal in 2010 [21, 22]. A meta-analysis of five randomized controlled trials utilizing GO combined with 7 + 3 for newly diagnosed patients demonstrated improved OS and risk of relapse compared to standard 7 + 3 alone with the greater share of benefit occurring in patients with favorable risk cytogenetics and no benefit demonstrated in patients with adverse cytogenetic characteristics [23]. GO was reapproved for treatment of newly diagnosed CD33 + AML in both induction and consolidation. However, NCCN recommends utilizing 7 + 3 + GO in cases of CBF-AML where allogeneic HSCT is not anticipated as consolidation in first complete remission (CR1) given the clearer benefit in this population based upon meta-analysis data and to mitigate liver toxicity from VOD associated with both GO and HSCT [24, 25].

### CPX-351

CPX-351 is a dual-drug liposomal formulation of 7 + 3 given as a discrete infusion of both drugs (typically given D1, D3, and D5 of induction) rather than requiring the continuous infusion of cytarabine over 7 days, which allows for less cumbersome administration both for healthcare providers and patients [26]. CPX-351 was initially studied in adults aged 60–75 with newly diagnosed AML, which demonstrated improved combined morphologic complete remission and complete remission with incomplete hematologic recovery (CR/CRi) (57.9% vs 31.6%) over 7 + 3 without statistically significant difference in OS or EFS. However, a planned subgroup analysis investigating CPX-351 in secondary AML (defined as AML with myelodysplasia related changes, AML with antecedent MDS or CMML, or therapy-related AML) demonstrated a signal of survival benefit in this population [27]. Therefore, a phase 3 study was conducted comparing CPX-351 vs 7 + 3 in patients aged 60–75 with newly diagnosed secondary AML (sAML), demonstrating improved median OS (9.56 vs 5.95 months) and CR/CRi (47.7% vs 33.3%) for CPX-351 with a similar safety profile [28]. Considering these results, CPX-351 is a preferred induction regimen for patients age > 60 with sAML who are candidates for intensive chemotherapy based on NCCN recommendations [25].

### Fludarabine/Cladribine-based Combinations

#### FLAG

Fludarabine is a purine analogue anti-metabolite chemotherapy with activity in chronic lymphocytic leukemia that has been showed to potentiate the effects of cytarabine by increasing the intracellular activity of the enzyme deoxycytidine kinase, which is responsible for activating cytarabine into its cytotoxic form [29]. Infusion of granulocyte-colony-stimulating factor (G-CSF) prior to fludarabine has also been shown to augment the accumulation of the active form of cytarabine [30]. This led to development of the FLAG regimen (fludarabine, cytarabine, G-CSF). A phase 2 study comparing FLAG to fludarabine + cytarabine (FA) as initial induction for AML (or MDS) resulted in a statistically similar CR rate (58% vs 45%, p = 0.50) and similar survival [31].

Because of the clinical benefit of high dose cytarabine in CBF-AML, a phase 2 study using FLAG induction and consolidation was performed in newly diagnosed CBF-AML which demonstrated an improved EFS over idarubicin and cytarabine ± G-CSF [32]. While FLAG has never been compared in a randomized fashion to 7 + 3, it is reasonable to consider this option for patients who are eligible for intensive treatment, but otherwise ineligible for anthracycline-based chemotherapy.

#### FLAG + GO

Motivated by promising results of FLAG in CBF-AML in combination with the demonstrated benefit of GO in CBF-AML, the regimen FLAG + GO was developed and studied in the induction and consolidation setting for this population [33]. Of 45 patients given FLAG-GO, 91% achieved CR with only 13% of patients relapsing at median follow up of 3 years [33]. Given the favorable prognosis in this group, median OS was not reached during the study, but an OS probability of 78% at 3 years was demonstrated [33]. Given these results, FLAG-GO has replaced FLAG as a regimen for patients with newly diagnosed CBF-AML and similarly is an option for fit patients for whom anthracycline is contraindicated.

#### FLAG-IDA ± GO

Building on the FLAG regimen, investigators added idarubicin to FLAG (FLAG-IDA) for induction of newly diagnosed disease. Small, phase 2 studies were conducted showing promising CR rates with reasonable toxicity [34], which lead to a large, phase 3 study including adults and children examining FLAG-IDA vs Daunorubicin + Cytarabine + Etoposide (ADE) [35]. Results from this study demonstrated a reduced relapse risk (38% vs 55%) favoring FLAG-IDA, as well as an improved relapse-free survival (RFS) without OS benefit [35]. In addition, FLAG-IDA has been combined with GO (FLAG-IDA + GO), demonstrating an significant survival benefit in CBF-AML without as clear benefit for intermediate-risk and adverse-risk cytogenetic groups, similar to previous studies examining GO [36].

#### CLAG-M

Like fludarabine, cladribine has a potentiating effect on cytarabine and has been studied in combination with G-CSF and mitoxantrone in both the relapsed/refractory setting and the newly diagnosed setting, demonstrating a similar CR rate (71%) as well as similar toxicity and duration of cytopenias to historical controls using 7 + 3 [37].

### Venetoclax-Based Regimens

#### FLAG-IDA + Venetoclax

Leveraging success with venetoclax in the intensive-treatment ineligible population, investigators have sought to incorporate venetoclax into regimens in the intensive treatment-eligible population as well. FLAG-IDA + Venetoclax (FLAG-IDA + VEN) is a newer induction regimen gaining traction due to an impressive ORR (CR + CRi + MFLS + PR) of 99% in newly diagnosed AML in a phase Ib/II study as well as preliminary results from the ongoing phase II study demonstrating a MRD negative rate of 89% and CR/CRi of 96%, despite 49% of patients having adverse-risk cytogenetics [38, 39]. It is worth noting that, while comprising a small subpopulation of the study, patients with mutant *TP53* appeared to do worse, with median duration of response (mDOR) of 8.2 months and mOS of 13.5 months [39].

#### Hypomethylating Agent + Venetoclax

While widely considered to be a preferred induction regimen for intensive treatment-ineligible patients with AML, azacitadine or decitabine plus venetoclax (HMA/ven) has been only recently brought into the intensive treatment-eligible patient population. PARADIGM, a multicenter phase 2 clinical trial randomizing newly diagnosed patients to either azacitadine and venetoclax (aza/ven) or 7 + 3 (or CPX-351 if indicated otherwise) which seeks to understand if HMA/ven in all patients with newly diagnosed AML regardless of fitness (Table 1). In addition, the VIALE-T trial is investigating use of aza/ven in the maintenance setting after HSCT (Table 1).

**Table 1:** Selected clinical trials in newly diagnosed AML

Intensive Chemotherapy Eligibility	Agent(s)	Phase of Treatment	Study Phase	NCT#
Eligible	Azacitadine + Venetoclax Or 7 + 3 (or CPX-351)	Induction	Phase 2	NCT04801797
Eligible	Venetoclax with 7 + 3	Induction/Consolidation	Phase 1b	NCT03709758
Eligible	Quizartinib with 7 + 3	Induction, Consolidation, Maintenance	Phase 3	NCT06578247
Eligible	Gilteritinib with 7 + 3 Or Midostaurin with 7 + 3	Induction, Consolidation, Maintenance	Phase 3	NCT03836209
Eligible	Crenolanib with 7 + 3 Or Midostaurin with 7 + 3	Induction, Consolidation, Maintenance	Phase 3	NCT03258931
Eligible	Ivosidenib or Enasidenib + SOC OR Placebo + SOC	Induction, Consolidation, Maintenance	Phase 3	NCT03839771
Eligible	7 + 3 + Midostaruin + Revumenib	Induction/Consolidation	Phase 1	NCT06313437
Eligible	7 + 3 + Revumenib	Induction/Consolidation	Phase 1b	NCT05886049
Eligible	7 + 3 + Revumenib	Induction/Consolidation	Phase 1	NCT06226571
Eligible or ineligible	7 + 3 + Ziftomenib Or Aza/Ven + Ziftomenib	Induction/Consolidation	Phase 1	NCT05735184
Eligible or ineligible	7 + 3 + Bleximenib Or Aza/Ven + Bleximenib	Induction/Consolidation	Phase 1	NCT05453903
Ineligible	Aza/Ven + Bleximenib	Induction/Consolidation	Phase 3	NCT06852222
Ineligible	Aza/Ven/Gilt	Induction/Consolidation	Phase 1/2	NCT05520567
Ineligible	Aza/Ven/Gilt	Induction/Consolidation	Phase 2	NCT06317649
Ineligible	Decitabine/Cedazuridine/Ven + Enasidenib	Induction/Consolidation	Phase 2	NCT06672146
Eligible	Azacitadine + Venetoclax	Maintenance (post-transplant)	Phase 3	NCT04161885
Eligible	Azacitadine (oral)	Maintenance (post-transplant)	Phase 3	NCT04173533

#### 7 + 3 + Venetoclax

Again, leveraging the success of venetoclax in the unfit population, there is also a phase 1b clinical trial actively enrolling patients for 7 + 3 + venetoclax induction followed by venetoclax in combination with standard consolidation in fit patients with newly diagnosed AML (Table 1).

#### CC-486 (Oral Azacitidine)

While many patients with AML are fit enough to undergo intensive chemotherapy with 7 + 3 or CPX-351, not all patients are candidates for allogeneic stem cell transplantation. Whether because of incompatible donor, progressive deconditioning, lack of social support, or inability to access a bone marrow transplant center due to transportation concerns, many patients are not able to undergo transplantation. Knowing that relapse risk is high in patients with intermediate-risk and adverse-risk cytogenetics without HSCT, CC-486 (oral azacitadine) was developed as an oral maintenance strategy and studied in a phase 3, randomized, placebo-controlled fashion in QUAZAR AML-001 [40]. Results from this trial demonstrated an improved median OS (24.7 vs 14.8 months) as well as improved relapse-free survival (10.2 vs 4.8 months) favoring CC-486 [40]. It is worth noting that most patients (80%) in this study received at least one cycle of consolidation chemotherapy [40]. These results led to FDA approval of CC-486 for patients eligible for intensive chemotherapy who were not suitable for allogeneic HSCT. There have been phase 2 data examining CC-486 as a maintenance strategy after HSCT, demonstrating estimated 81% survival at one year post transplant [41]. However, the phase 3 study comparing CC-486 maintenance to placebo post-transplant is ongoing (Table 1).

### FLT3-Mutated AML

FMS-like tyrosine kinase 3 (FLT3) is a receptor tyrosine kinase present on normal hematopoietic stem cells and interacts with its ligand (FL) produced by bone marrow stroma cells to promote survival, proliferation, and differentiation in the normal bone marrow environment [42]. Mutations in *FLT3* result in exaggerated survival and proliferation of hematopoietic stem cells and is a relatively common mutation in AML (approximately one-third) [43]. There are two *FLT3* mutations of clinical significance, *FLT3* with internal tandem duplication (*FLT3*-ITD) and tyrosine kinase domain mutated *FLT3* (*FLT3*-TKD) [44]. There are currently three FLT3 inhibitors FDA approved for use in AML (midostaurin, quizartinib, gilteritinib), each with their own treatment niches.

Midostaurin is an oral tyrosine kinase inhibitor (TKI) with activity against *FLT3*-TKD and *FLT3*-ITD mutations. Promising pre-clinical and early phase clinical trials in the relapsed/refractory setting led to the multicenter, placebo-controlled randomized phase 3 trial RATIFY investigating 7 + 3 with midostaurin induction in patients with newly-diagnosed *FLT3*-mutated AML (both TKD and ITD mutations included) [45]. Median OS was 74.7 months in the midostaurin group vs 25.6 months in the placebo group [45]. As part of the protocol, patients achieving remission after induction were consolidated with four cycles of HiDAC and midostaurin or placebo followed by midostaurin or placebo maintenance for twelve cycles. Allogeneic HSCT was not required as part of the trial protocol regardless of cytogenetic risk. The results of RATIFY cemented midostaurin (and FLT3 inhibition broadly) as an integral addition to induction chemotherapy for newly diagnosed *FLT3*-mutated AML.

Quizartinib is a newer generation TKI with activity against *FLT3*-ITD mutated AML. Quizartinib inhibits the inactive form of the protein rather than the active form [46]. As a result of its mechanism, quizartinib is not thought to have activity against TKD mutations. Success of quizartinib in early phase trials led to the randomized, placebo-controlled phase 3 trial QuANTUM-First investigating quizartinib in induction (with 7 + 3), consolidation (with HiDAC), and maintenance for patients with newly diagnosed *FLT3*-ITD mutated AML. Median OS in the quizartinib group was found to be 31.9 months versus 15.1 months in the control group [46]. Given the results of these two trials, both midostaurin and quizartinib are FDA approved for use in newly diagnosed *FLT3*-ITD AML for patients fit for intensive chemotherapy, with only midostaurin being approved for *FLT3*-TKD mutated AML. There have been no head-to-head prospective comparisons of midostaurin and quizartinib. However, there are some real-world data that have retrospectively compared these two agents, demonstrating a signal of possible benefit for quizartinib, probably owing to the overall better side effect profile resulting in fewer dose interruptions [47]. In this study, CRc rates were higher with quizartinib than with midostaurin (85% vs 73%) with similar rates of febrile neutropenia, infections, need for parenteral nutrition, G-CSF use, and ICU transfers [47]. Given quizartinib’s potential activity against wild-type FLT3, there is a study investigating its use in newly diagnosed AML without *FLT3* mutation that is actively recruiting (Table 1).

Sorafenib is a multitargeted TKI that has been demonstrated to have activity in *FLT3*-ITD mutated AML in addition to other cancer types such as renal cell carcinoma, hepatocellular carcinoma, and thyroid cancer [48]. Several studies postulated that sorafenib could reduce risk of relapse after allogeneic HSCT in *FLT3*-ITD mutated AML, leading to a randomized, placebo-controlled, phase 3 trial to test this hypothesis [48]. Median relapse-free survival (RFS) was not reached in the sorafenib group at 41.8 months, while noted to be 30.9 months in the placebo group [48]. However, sorafenib has not been compared head-to-head with either midostaurin or quizartinib and is not presently FDA approved for AML in the United States.

Gilteritinib is a type 1 FLT3 inhibitor with activity against both *FLT3*-TKD and *FLT3*-ITD and has been established as an active agent in the relapsed/refractory setting for *FLT3*-mutated AML [49]. Early phase studies in the newly diagnosed setting combining gilteritinib with intensive AML therapy suggest activity that the drug is active in this context [50], though studies comparing gilteritinib to midostaurin are ongoing (Table 1). Early results suggest higher rates of CRc with gilteritinib (85.6% vs 72.4%) with survival data yet to be reported [51].

Crenolanib is an investigational type 1 FLT3 inhibitor with activity against FLT3-TKD and FLT3-ITD with the added benefit of not inhibiting KIT, a feature that is postulated to attenuate the myelosuppression seen with other FLT3 inhibitors [52]. A phase 2 study was conducted combining crenolanib with 7 + 3 for newly diagnosed FLT3-mutated AML, demonstrating CRc rate of 86% with median OS not reached at 45 months follow-up [52]. A phase 3 trial comparing crenolanib to midostaurin is underway (Table 1). Crenolanib has also been studied in the post-HSCT setting as maintenance with encouraging results [53].

#### IDH1/2-Mutated AML

Isocitrate dehydrogenase 1 and 2 are enzymes involved in the citric acid cycle and are responsible for conversion of D-isocitrate to alpha-ketoglutarate, generating NADH from NAD + in the process [54]. Mutated *IDH1* or *IDH2* results in a reduction of this expected reaction and promote a reaction converting alpha-ketoglutarate to 2-hydroxyglutarate (2HG), whose intracellular accumulation leads to downstream epigenetic changes resulting in block of myeloid differentiation [54]. Breakthroughs in understanding the pathophysiology driving *IDH1* and *IDH2* mutated AML led to development of oral small molecule inhibitors targeting these mutated enzymes, ivosidenib (IDH1), olutasidenib (IDH1), and enasidenib (IDH2). These drugs have largely been used in newly diagnosed patients who are unfit for intensive chemotherapy and patients with relapsed/refractory disease. However, these agents are under investigation for newly-diagnosed, fit patients as well.

Ivosidenib has been studied as post-transplant maintenance for *IDH1*-mutated AML, with phase 1 data demonstrating encouraging safety data and two year OS of 88% [55]. Enasidenib was also studied in the post-transplant maintenance setting with good safety data as well and two year OS of 74% [56]. Both ivosidenib and enasidenib have also been studied in combination with intensive chemotherapy in both induction and consolidation phases of treatment with a phase 1 study [57]. In this study, Composite CR rates were 72% and 63% with ivosidenib and enasidenib, respectively with reasonable safety data for both, with safety profiles closely representing that of intensive chemotherapy alone [57]. There is an international, double blind, randomized, phase 3 trial that is currently investigating adding ivosidenib or enasidenib versus placebo in combination with intensive induction chemotherapy (eg, 7 + 3), consolidation, and maintenance for *IDH1* or *IDH2* mutated newly diagnosed AML and MDS-EB2 (Table 1).

### *KMT2A* and/or *NPM1*-mutated AML

*Lysine methyltransferase 2A* (*KMT2A*) rearrangements are present in about 10% of acute leukemias and lead to aberrant expression of homeobox genes, resulting in myeloid differentiation block, by interacting with menin in a high-affinity manner [58]. *Nucleophosmin 1* (*NPM1*) is a much more commonly mutated gene in AML (approximately 30%) and interacts with wild-type KMT2A and menin to block myeloid differentiation and drive leukemogenesis similarly by following the same epigenetic pathway [58]. There are several menin-inhibitors currently under investigation: revumenib, bleximenib, ziftomenib, enzomenib, emilumenib, BN-104, and BMF-219. Revumenib is currently the only menin-inhibitor that is FDA approved. This approval is for the relapsed/refractory *KMT2A*-rearranged (*KMTRAr*) population and is based on the results of the AUGMENT-101 study. In the *KMT2Ar* portion of this study, patients with relapsed/refractory *KMT2Ar* AML were treated with revumenib in a phase I/II fashion [59]. This population achieved an ORR (CR + CRh + CRi + CRp + MFLS + PR) of 63.2% and a CR/CRh rate of 22.8%. Of note, patients had largely been heavily pretreated (45% of patients having received 3 or more prior lines of therapy), with major adverse events being febrile neutropenia (37.2%), differentiation syndrome (16.0%), and QTc prolongation (13.8%) [59]. There is one phase 1 trial investigating revumenib in combination with midostaurin plus intensive induction chemotherapy and consolidation for newly diagnosed patients with *FLT3* and *NPM1* mutated AML (Table 1). Revumenib is also being studied as a pre-emptive therapy for MRD relapse after first or second complete remission (CR1/CR2). Early data from this study have shown MRD reduction or even MRD negativity in 5/8 patients with *NPM1m* AML, with accrual in the *KMT2Ar* population ongoing [60]. Additionally, ziftomenib and bleximenib are currently being studied in combination with either 7 + 3 or aza/ven in fit or unfit patients, respectively (Table 1). Interim results presented at the European Hematology Association (EHA) 2025 Congress from the KOMET-007 study investigating ziftomenib in combination with 7 + 3 induction show 32/35 (94%) of patients with *NPM1*-mutated AML achieving CRc and 10/16 (83%) of *KMT2A*-rearranged patients achieved CRc [61]. Interim results from the bleximenib study have demonstrated ORR of 93%, with a CR/CRh rate of 86% [62]. Mature survival data from each of these studies have not yet been reported.

## Options for Intensive Treatment-Ineligible Patients

### AML without Targetable Genetic Aberration

Older, unfit adults treated with intensive chemotherapy (e.g. 7 + 3) historically have had poor outcomes, with 5-year OS rates of less than 10–15% [63]. To address the unmet need in elderly AML, several regimens have been developed with the intent of balancing efficacy and toxicity.

#### LDAC-Based Regimens

Since the 1970s, Low-Dose Cytarabine (LDAC) has been used in the treatment of older, unfit patients with AML due to improvement in transfusion-independence, and later studies demonstrated a reasonable CR rate of 31% [64, 65]. LDAC was then compared via randomized, phase 3 trial to intensive chemotherapy, which demonstrated no difference in overall survival, but the LDAC group required fewer blood products, had fewer infectious complications, and had fewer early deaths [66]. With the advent of newer oral therapies such as glasdegib and venetoclax, LDAC has been used as a backbone of cytotoxic chemotherapy in combination with these agents in a much similar fashion to 7 + 3 in fit patients.

Glasdegib is an oral hedgehog-pathway inhibitor which acts by blocking smoothened, the receptor for sonic hedgehog. The hedgehog pathway is an embryonic signaling pathway that promotes differentiation during early embryo development. This pathway is typically silenced in adults, but abnormalities in this pathway have been implicated in causing chemoresistance in hematologic malignancies including AML [67, 68]. For this reason, glasdegib has been studied in combination with LDAC and is FDA approved for newly diagnosed AML in unfit, older adults. This approval is based on results demonstrating mOS difference of 8.3 months vs 4.3 months as well as CR rates of 19.2% vs 2.6%, both favoring LDAC + glasdegib over LDAC alone [69].

LDAC has also been combined with Venetoclax in a phase 3 randomized, placebo-controlled study comparing LDAC + ven to LDAC alone. Results from this study demonstrated mOS benefit of 8.4 months to 4.1 months as well as CR/CRi benefit of 48% vs 13% and greater transfusion independence in the venetoclax arm (37% vs 17%) [70].

#### Gemtuzumab Ozogamicin

As discussed earlier, GO received FDA approval initially for relapsed AML, but the approval was later withdrawn [20, 21]. After success using GO in combination with 7 + 3 in fit patients with CD33 + CBF-AML, investigators sought to use GO again in the older adult, unfit population.

GO was compared to best supportive care in a randomized, phase 3 study, with a modest mOS benefit favoring GO of 4.9 vs 3.6 months [71]. Perhaps more significant, was an estimated 2-year survival of 24.3% vs 9.7% favoring GO [71]. While available for newly diagnosed unfit patients with AML, GO is not generally considered to be the best option for such patients, due to the successes of other regimens such as aza/ven.

### Hypomethylating Agent-Based Regimens

#### Single Agent Azacitadine or Decitabine

Given efficacy demonstrated in MDS, investigators sought to study HMAs for newly diagnosed AML in unfit patients. Initially, azacitidine was studied in unfit patients with low blast count AML (defined as 20%-30% bone marrow blasts). A phase III study comparing single agent azacitidine to conventional-care regimens (CCRs) including BSC, LDAC, and intensive chemotherapy demonstrated mOS benefit of 24.5 months in the azacitidine compared with 16.0 months in the CCR group [72]. Azacitidine was then studied in unfit patients with > 30% blast count in the bone marrow in a phase III study compared to CCR, which demonstrated a mOS benefit of 10.4 months vs 6.5 months with statically equivalent CR + CRi rates (~ 25%) [12].

Similarly, decitabine was studied in comparison to patient’s choice (BSC or LDAC) in a phase III randomized study in unfit patients newly diagnosed AML and results were similar to the studies investigating single agent azacitadine, though the findings with decitabine were not statistically significant compared to control [13]. Median OS was 7.7 months in the decitabine arm vs 5.0 in PC arm, while CR + CRi rates were 25.6% vs 10.3%.

#### HMA/Venetoclax-based Regimens

Venetoclax was first studied in AML in the 2010s after efficacy was demonstrated in CLL. A phase II study was conducted investigating use as single agent in unfit patients with newly diagnosed AML as well as relapsed/refractory AML [73]. This study demonstrated a CR + CRi rate of 19% with reasonable safety data, most notable for a neutropenic fever rate of 28% [73].

Due to a hypothetical synergistic effect, azacitidine and venetoclax (aza/ven) as well as decitabine and venetoclax were studied in combination in a phase Ib study demonstrating a CR + CRi rate of 71% and mOS of 16.9 months [74, 75]. These promising results led to the landmark VIALE-A phase III randomized trial comparing aza/ven to azacitidine alone in unfit patients with newly diagnosed AML [14]. In this study, aza/ven was shown to have a statistically significant clinical benefit over azacitidine alone, including mOS advantage of 14.7 months vs 9.6 months as well as a CR + CRi benefit of 66.4% vs 28.3% with similar toxicity seen with exception of higher incidence of febrile neutropenia [14]. This study has established aza/ven as the de facto standard of care treatment for newly diagnosed, unfit patients with a NCCN Category 1 recommendation[25]. Decitabine and venetoclax has not been studied in the phase III setting, but data in earlier phase studies has garnered support for its use.

Oral decitabine/cedazuridine has been combined with venetoclax as well in newly diagnosed unfit patients. Cedazuridine is a cytidine deaminase inhibitor that prolongs the half-life of decitabine, allowing for use in an oral formulation. A phase 2 study combining decitabine/cedazuridine with venetoclax was published in 2024 demonstrating an ORR of 64% in newly diagnosed patients [76]. Phase III data is needed to establish this regimen as a preferred regimen along with Aza/Ven. However, the benefit of this regimen is that it is a completely oral regimen and is a good option for patients who cannot reliably receive infusion five (or seven) days consecutively.

There has been a phase II study investigating two courses of cladribine plus LDAC in combination with venetoclax alternating with two courses of aza/ven to see if alternating therapies can reduce resistance mechanisms and cumulative toxicity [77]. CR + CRi rates were found to be 93% (CR rate 80%) with MRD negativity after induction noted to be 84% [77]. Survival data were encouraging, with median OS not reached at median follow up of 22.1 months [77].

### FLT3-Mutated AML

As discussed previously, gilteritinib has been shown to have activity in relapsed/refractory AML [49], which has led to studies investigating the addition of gilteritinib to azacitadine (aza/gilt) in newly diagnosed patients with *FLT3*-ITD mutated AML unfit for intensive chemotherapy. Aza/gilt was compared to azacitidine alone in a phase III, randomized (though not placebo-controlled) fashion in the LACEWING study [78]. Median OS was not found to be statistically different in the two groups, with the aza/gilt arm having a mOS of 9.82 months vs 8.87 months in the single agent azacitidine arm [78]. The lack of survival difference may be influenced by the lack of a placebo in the control group, thus permitting patients on the azacitidine arm to come off study and receive other therapies. 28.6% of patients in the aza alone arm received a subsequent FLT3 inhibitor, compared to 4.1% of patients in the aza/gilt arm. A subgroup analysis did reveal a signal of benefit in the high allelic burden *FLT3* (allelic ratio > 0.5) subgroup, with aza/gilt having a mOS of 10.68 months vs 4.34 months in the azacitidine alone group [78].

Investigators have also examined combining azacitadine, venetoclax, and gilteritinib (aza/ven/gilt) for newly diagnosed, *FLT3*-mutated patients. A combined phase 1/2 study at MD Anderson Cancer Center investigating this combination was performed both in newly diagnosed patients and patients with relapsed/refractory disease [79]. This study demonstrated a CRc rate of 96% (CR rate 90%) in newly diagnosed patients with mOS not reached at a median follow up of 19.3 months [79]. These results are promising, and there is an ongoing larger phase 2 study testing this regimen as part of the MyeloMATCH program (Table 1).

### *IDH1/2*-Mutated AML

After demonstrating success in relapsed/refractory *IDH1* mutated AML as a single agent, investigators sought to combine ivosidenib with azacitidine (aza/ivo) in newly diagnosed, unfit patients. A phase 3, randomized trial comparing aza/ivo to azacitidine was conducted, demonstrating a mOS benefit of 24.0 months with aza/ivo over 7.9 months with azacitadine alone, which has led to this regimen becoming a standard of care treatment for newly diagnosed *IDH1*-mutated patients [80]. However, aza/ivo has not been compared head-to-head to HMA/ven.

The combination of ivosidenib added to aza/ven has been studied in the phase 1b/2 setting in newly diagnosed and R/R patients as well as patients with *IDH1*-mutated MDS or an *IDH1* mutated MPN, demonstrating an impressive 93% CRc rate and 86% MRD negative rate [81].

Similar to aza/ivo, azacitidine and enasidenib (aza/ena) has been studied in newly diagnosed, *IDH2*-mutated unfit patients after strong results in the relapsed/refractory setting. However, there has not been published data in the phase 3 setting for Aza/Ena. There was a phase 2 study that randomized patients to aza/ena vs aza alone demonstrating a CRc rate of 66% in the aza/ena group and mOS of 22.0 months compared with mOS of 22.3 months in the aza alone group [82]. Like LACEWING in the *FLT3* mutated population, the survival data from this study may have been influenced by the lack of placebo in the control group. Similarly to aza/ivo, aza/ena has not been compared to aza/ven directly in a randomized fashion.

### *KMT2A*-rearranged and/or *NPM1*-mutated AML

There have been two small scale studies investigating revumenib in combination with HMA/ven for upfront treatment of newly diagnosed, *KMT2A*-rearranged and/or *NPM1*-mutated AML in patients unfit for intensive therapy. Of 13 patients treated with aza/ven plus revumenib, all 13 had an objective response and 10 patients achieved CR [83]. Revumenib has also been added to decitabine/cedazuridine and venetoclax as an all oral regimen, with 9/9 patients achieving objective response and 7/9 patients achieving CR/CRi/CRp [84]. The results of these studies are quite promising, though more, larger studies are needed to prove clinically significant efficacy compared to current standards of care. As previously mentioned, ziftomenib and bleximenib are being investigated in combination with either 7 + 3 (fit) or aza/ven (unfit) in newly diagnosed patients, with the studies actively recruiting (Table 1). Phase Ib data was presented at EHA Congress in June 2025 investigating aza/ven/bleximenib as induction as well as salvage therapy in unfit patients with CRc rate of 85% and ORR of 92% at 100mg BID of bleximenib in the newly diagnosed population [85]. In addition, emilumenib (DS-1594) is being studied in a phase I/II trial in combination with aza/ven in newly diagnosed unfit patients, with data yet to be reported. KOMET-017 is a phase 3 randomized trial comparing standard of care induction therapy in combination with ziftomenib versus placebo in both fit and unfit patients, and is expected to start enrolling in the summer of 2025.

## Conclusion

In conclusion, the management of newly diagnosed AML differs greatly between patients eligible for intensive chemotherapy versus patients who are ineligible. However, there are some commonalities between the two disease groups with regards to advances made in the last two decades. Gemtuzumab ozogamicin is an option for fit and unfit patients who have CD33 expressing blasts, with greatest benefit being observed in CBF-AML. HMA/ven is showing such success in the unfit population that it is challenging our traditional paradigm of 7 + 3 in patients who are fit for intensive chemotherapy. The advent of small molecule inhibitors in tandem with increased accessibility to next generation sequencing has promoted the ongoing development and use of therapies in both fit and unfit patients as well. More studies are needed to determine optimal sequencing, but results of FLT3, IDH1, IDH2, and menin inhibition in frontline treatment are beginning to establish new standards of care in the field. The combination of targeted small molecule inhibitors with the HMA/ven backbone promises to improve remission rates and hopefully survival in AML. In addition, the advent of targeted therapies raises the question of utilizing these agents in the post-transplant setting to prevent relapse, as relapse occurs in approximately 20–30% of AML patients at five years after allogeneic transplant and is the greatest cause of mortality following transplantation [86].

## Key References

- Dinardo et al. Azacitidine and Venetoclax in Previously Untreated Acute Myeloid Leukemia. New England Journal of Medicine. 2020;383(7):617–29
  o This reference is of importance because it established azacitadine and venetoclax as standard induction treatment for newly diagnosed patients with AML considered unfit for intensive induction chemotherapy. This study has inspired a phase III clinical trial comparing the regimen to traditional “7 + 3” induction chemotherapy in fit patients as well.
- Montesinos et al. Ivosidenib and Azacitidine in IDH1-Mutated Acute Myeloid Leukemia. New England Journal of Medicine. 2022;386(16):1519–31
  o This reference is of importance for establishing azacitadine and ivosidenib as the standard induction treatment for IDH1-mutated AML in unfit patients.
- Erba et al. Quizartinib plus chemotherapy in newly diagnosed patients with FLT3-internal-tandem-duplication-positive acute myeloid leukaemia (QuANTUM-First): a randomised, double-blind, placebo-controlled, phase 3 trial. Lancet. 2023;401(10388):1571–83
  o This reference is of importance for demonstrating overall survival benefit of adding quizartinib to standard intensive induction chemotherapy in fit patients with FLT3-ITD mutated AML.

## Data Availability

No datasets were generated or analysed during the current study.
